# Role of Cerebral Oximetry in Reducing Postoperative End-Organ Dysfunction After Major Non-Cardiac Surgery: A Randomised Controlled Trial

**DOI:** 10.3390/clinpract15110213

**Published:** 2025-11-18

**Authors:** Matthanja Bieze, Karen Foley, W. Scott Beattie, Jo Carroll, Humara Poonawala, Lian-Kah Ti, George Djaiani

**Affiliations:** 1 Department of Anesthesia & Pain Management, University of Toronto, Toronto General Hospital, University Health Network, Toronto, ON M5G 2C4, Canada; 2 Department of Anesthesia, Yong Loo Lin School of Medicine, National University of Singapore, Singapore 117597, Singapore

**Keywords:** non-cardiac surgery, cerebral oximetry, major organ morbidity and mortality, postoperative complications, anaesthesia

## Abstract

**Background/Objectives**: An increasing number of older individuals require general anaesthesia for major non-cardiac surgery, with 20% displaying postoperative complications. Regional cerebral oxygen saturation (rSO_2_) correlates with the gold standard of mixed venous oxygen saturation, indicating global perfusion. We hypothesised that rSO_2_-based anaesthesia reduces organ dysfunction and morbidity after major non-cardiac surgery. **Methods**: In Singapore and Toronto, we conducted a prospective, double-blind, randomised controlled trial in elderly patients undergoing major non-cardiac surgery, after obtaining research ethics board permission and informed consent. This RCT followed the CONSORT guidelines. Patients received bilateral cerebral oximetry sensors, and the control group received standard care. In the intervention group, an algorithm restored rSO_2_ if it dropped 10% below baseline for >15 s by adjusting cerebral perfusion pressure, inspired oxygen concentration, end-tidal carbon dioxide, depth of anaesthesia, haemoglobin, and cardiac index. Postoperative complications and outcomes were noted. Categorical data were analysed using Chi-square or Fisher’s exact tests and continuous data using a *t*-test or a Mann–Whitney U test. The study was powered for 394 patients, but due to the COVID-19 pandemic and funding constraints, this study was terminated at 101 patients. **Results**: Of 101 patients, 49 were randomised to the control and 52 to the intervention group. A total of 31 (63%) patients in the control group and 30 (58%) in the interventional exhibited bilateral cerebral desaturation. Time of cumulative cerebral desaturation was longer in the control group (23 ± 48 min vs. 9 ± 15 min, respectively, *p* = 0.01). A total of 142 algorithm-based treatments were employed, restoring rSO_2_ in 29 (86%) patients. Both groups displayed equal postoperative outcomes. **Conclusions**: In major non-cardiac surgery, cerebral desaturation is prevalent in over 85% of patients. Although algorithm-guided therapy restored rSO_2_ in the majority of patients, it did not result in reduced postoperative morbidity.

## 1. Introduction

With an increase in life expectancy observed over the last two decades, the number of elderly patients requiring general anaesthesia for major non-cardiac surgery has increased dramatically. One of the largest prospective studies of over 4000 elderly patients undergoing major non-cardiac surgery identified that 68% of these patients had pre-existing comorbidities. Furthermore, the 30-day postoperative mortality was 5%, and major postoperative complications were reported in 20% of patients [1].

The primary focus of intraoperative patient management is to ensure adequate perfusion and oxygen delivery to the vital organs. Continuous non-invasive monitoring of regional cerebral oxygen saturation (rSO_2_) through the application of near-infrared spectroscopy technology provides information on the real-time status of the balance between brain oxygen supply and demand [2]. Furthermore, it provides extra assurance of the adequacy of global oxygen balance, particularly focusing on the venous side of circulation [3]. With the current standards of monitoring that primarily focus on the left heart, such as oxygen supply, and not the imbalance between oxygen supply/demand, vital organ ischemia may go unnoticed until functional organ damage becomes clinically evident. The cerebral oximeter provides a non-invasive alternative method of measuring the adequacy of systemic oxygen balance that correlates well with the gold standard of mixed venous oxygen saturation [4,5,6].

It has been previously reported that different levels of cerebral desaturation can be detected in one out of four patients during non-cardiac surgery [7,8]. Decreased rSO_2_ has been associated with postoperative cognitive dysfunction [9,10,11], perioperative stroke [12], increased incidence of major organ morbidity [2], and 30-day and 1-year mortality [13]. Furthermore, decreased preoperative rSO_2_ measurements have been associated with a higher risk of postoperative delirium in both cardiac [14] and non-cardiac [15] surgical populations. In non-cardiac surgical patients, an association was found between decreased rSO_2_, postoperative complications, delay in recovery room discharge, and prolonged hospital length of stay [8,16]. However, it is less clear whether interventions designed to reverse or minimize rSO_2_ reduction would result in decreased perioperative morbidity and mortality. Consequently, the primary aim of the current study was to determine whether restoration of rSO_2_ resulted in reduced incidence of composite morbidity and improved functional recovery after major non-cardiac surgery.

## 2. Materials and Methods

We conducted a prospective, two-centre, randomised, double-blind, controlled clinical trial from January 2019 (Toronto) and November 2019 (Singapore) to June 2023. The study was conducted in accordance with the Declaration of Helsinki and approved by the Institutional Review Board of the University of Toronto (CAPCR 16-5747.8); the REB underwent multiple renewals. NHG Domain Specific Review Board approval was granted for the period from 14 November 2019 to 13 November 2020 for the trial to be conducted in the National University Hospital, Singapore. The NHG Domain Specific Review Board reference number for this study is 2019/00827. The termination date for patient recruitment at the Singapore site was as per the initial IRB approval. Singapore did not recruit any patients beyond 13 November 2020. The renewal IRB for Singapore was only for data extraction and analysis.

This study was conducted in quaternary referral hospitals in Toronto, Canada, and Singapore. The current trial was registered on 5 October 2018, at www.clinicaltrials.gov, with the unique identifiers NCT 03861026 (Toronto) and NCT 04627506 (Singapore). This RCT followed the CONSORT guidelines.

The study group comprised patients 60 years of age or older undergoing elective major non-cardiac surgery (a significant surgical procedure that involves high risk and requires general anaesthesia, a hospital stay, and a long recovery period. Examples include total abdominal hysterectomy, DIEP, and liver resection), with an anticipated surgical duration of at least 3 h. We recruited patients through the anaesthesia pre-admission clinic, and patients provided written, informed consent. Patients were excluded if they required emergency surgery, laparoscopic or robotic surgery, or if they exhibited serious mental illness, severe dementia, delirium, or were unable to sign the consent form.

Randomisation occurred prior to surgery using a computer-generated randomisation code in permuted blocks of four. Bilateral cerebral oximeter sensors (Masimo, Root^®^ O3^®^ Regional Oximetry) were placed on the frontotemporal regions in all patients, and baseline values of rSO_2_ on both the right and left sides were obtained and recorded in the operating room prior to induction of anaesthesia.

In the intervention group, an alarm threshold at 90% of the baseline rSO_2_ value was established such that if rSO_2_ reduced 10% below baseline for >15 s, the intervention algorithm was initiated. The algorithm included an increase in cerebral perfusion pressure > 70 mmHg (mean arterial pressure ≥ 65 mmHg and jugular venous pressure < 10 mmHg), an increase in inspired O_2_ to 100%, an increase in the depth of anaesthesia to decrease cerebral oxygen consumption, an adjustment of PaCO_2_ or end-tidal CO_2_ ≥ 40 mmHg, red cell transfusion if the haematocrit was <20%, and an increase in cardiac index to ≥2 L/m2/min if cardiac output measurement devices were used. There was no stepwise strategy to the proposed algorithm, more than one intervention was allowed at any one time, and there were no actual physiological target endpoints were quantified for individual patients other than increasing the rSO_2_ value.

In the control group, the cerebral oximetry monitor screen was concealed after the signal strength and baseline values were acquired by the independent observer trained in cerebral oximetry application, who was also unaware of the study design.

Anaesthesia and surgical management were conducted according to routine institutional practice. Standard Canadian Anaesthesia Society monitoring was employed. In addition, all patients received arterial and central venous pressure lines. The use of cardiac output measurement devices was left to the discretion of the anaesthesiologist in charge of the case. Anaesthesia was induced with fentanyl and propofol. Tracheal intubation was facilitated with rocuronium. Maintenance of anaesthesia was achieved with sevoflurane and supplemented with fentanyl, hydromorphone, and rocuronium. Depth of anaesthesia was measured using a Masimo SEDLine™ device, and patient state index was maintained in the range of 25 to 50. Postoperative analgesia was provided through opioid analgesics and non-opioid adjuvants, as per standard clinical practice.

The primary outcome of the study was predetermined as a composite outcome defined as major organ morbidity and mortality. These outcomes included postoperative delirium (assessed by confusion assessment method at baseline and on postoperative days 1 to 3), stroke (any functional deficit exceeding a 24 h period), transient ischemic attacks (any functional deficit for less than a 24 h period), myocardial infarction (based on the Fouth Universal Definition of Myocardial Infarction), pulmonary embolism (clinical, echocardiography, and computer tomography assessments), renal failure (creatinine increase ≥ 50% from baseline), pneumonia (clinical, X-ray, microbiology assessment), re-intubation within 24 h, atrial fibrillation (documented via electrocardiogram), major bleeding (requiring ≥4 units of red cell transfusion within 72 h of surgery), mechanical ventilation for ≥48 h after surgery, major wound disruption, surgical site wound infection, sepsis (positive blood cultures, antibiotics, identified source of infection, vasoactive medications), unplanned return to the operating room, and all-cause 30-day mortality. The secondary outcomes included postoperative Quality of Recovery-15 (QoR-15) scores, Disability Free Survival (DFS) at 6 months (World Health Organization Disability Assessment; WHODAS), and hospital length of stay.

Given the 20% prevalence of a composite outcome and in order to obtain a 50% reduction in major morbidity and mortality rates in patients receiving the rSO_2_ restoration algorithm management strategy, with α = 0.05 and power 1 − β = 0.8, a group of 197 patients in each arm of the study was required, comprising total of 394 patients in the randomisation schedule. Descriptive statistics were used for all variables before and after surgery. Continuous data was presented as a mean with standard deviation or a median with range. Proportions were used for categorical variables. Continuous normally distributed data was analysed using a two-tailed Student’s *t*-test. The Mann–Whitney U test was used for skewed data. The Chi-square test or Fishers Exact test were used for categorical data. *p* < 0.05 was considered statistically significant. Statistical analysis was conducted through the use of MINITAB^®^ 18 statistical software (Minitab Inc., State College, PA, USA).

## 3. Results

### Outcomes

**Figure 1:**
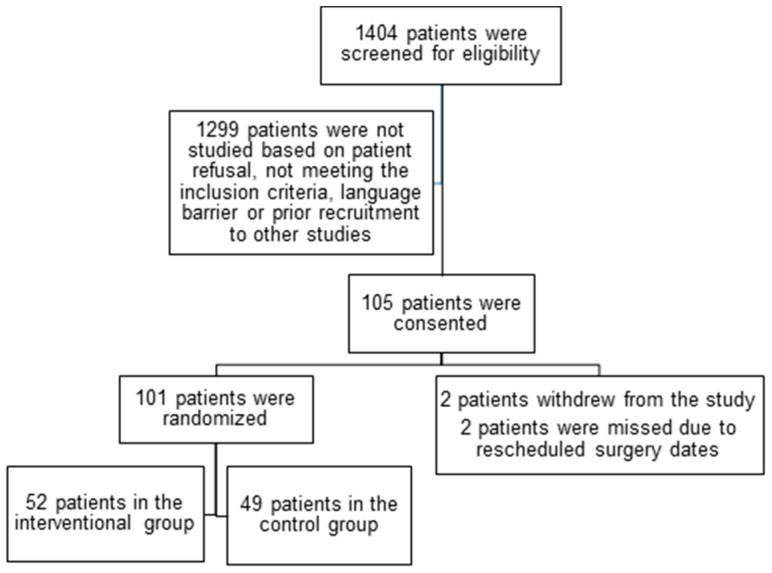
CONSORT diagram of the study.

A total of 1404 subjects were screened for eligibility, and 1299 were not studied due to patient refusal, failure to meet the inclusion criteria, a language barrier, or prior recruitment to other studies. A total of 105 patients consented. Two patients withdrew their consent, and two patients were lost due to a change in their surgery dates. Although a priori sample size calculations required a total of 394 patients, enrolment into the study was halted early due to the COVID-19 pandemic and funding constraints. Consequently, a total of 101 patients were randomised, with 52 and 49 patients in the interventional and control groups, respectively (Figure 1). Both groups were similar with respect to baseline demographic data, pre-operative medications, comorbidities, and surgical characteristics (Table 1).

Baseline cerebral oximetry data, as well as the incidence and duration of cerebral desaturation, are reflected in Table 2. There was no difference with respect to the incidence or maximum reduction in desaturation between the interventional and control groups, respectively. The median duration of bilateral desaturation was 5 (range 0 to 128) minutes vs. 9 (range 0 to 364) minutes in the interventional and control groups, respectively; *p* = 0.01.

In the interventional group, the algorithm led to a total of 142 interventions with a median of two interventions per patient, ranging from 1 to 12. A total of 32 (61.5%) patients received either volume administration and/or vasoactive medications to maintain adequate cerebral perfusion pressure. A total of 18 (35%) patients displayed an increase in inspired oxygen concentration, 11 (21%) patients showed adjustments in end-tidal or arterial CO_2_, 12 (23%) patients exhibited an increase in the depth of anaesthesia, and 5 (10%) patients received red cell transfusion. The restoration of rSO_2_ in the interventional group was achieved in 45 of 52 (86.5%) patients.

Postoperative morbidity was similar between the two groups, as shown in Table 3. None of the patients had died at 30-days in either group. A total of 8 (15%) patients in the interventional group and 7 (14%) patients in the control group developed postoperative complications. Most of these patients exhibited more than one complication each. The quality of recovery scores was significantly lower on postoperative day 1 and at discharge in both groups of patients when compared to the baseline; however, there was no difference in any of the functional scores between the two groups, as seen in Table 3.

## 4. Discussion

The current study sought to determine whether the interventions designed to restore regional cerebral oxygen desaturation events would result in decreased perioperative major organ dysfunction and improved functional recovery after major non-cardiac surgery. A drop in rSO_2_ of more than 10% from the baseline value occurred in more than half of patients. The restoration of rSO_2_ was achieved in 86.5% of patients. In addition, even though the duration of the cumulative desaturation burden was significantly longer in the control group, major postoperative morbidity and functional recovery were similar between the two groups.

Different rSO_2_ reduction thresholds in the range of 10% to 25% have been previously utilized by our own research group, as well as by other investigators, to determine optimal timing for intervention. The success rates in restoring rSO_2_ to baseline values varied from 70% to 97% [17,18,19]. In the current study, we intentionally chose a threshold of a 10% reduction in rSO_2_ from baseline to trigger the intervention arm early in order to minimize the potential for end-organ hypoperfusion. It is important to note that the algorithm used in the interventional arm of the study included multiple physiological and pharmacological adjustments to reverse rSO_2_ reduction. However, the intervention rates, including blood product transfusion, inotropic support, and adjustments in ventilatory parameters, were similar between the two groups. It is possible that such an rSO_2_ drop of 10% was not sensitive enough compared to the standard parameters to trigger earlier intervention or that a 10% reduction in rSO_2_ alone was insufficient to cause end-organ hypoperfusion. This contention is supported by the recent American Society for Enhanced Recovery and Perioperative Quality Initiative joint consensus statement that states that there is insufficient evidence to recommend using intraoperative cerebral oximetry to improve outcomes after both cardiac and non-cardiac surgery [20].

However, this statement was recently challenged by a large retrospective review of 1.2 million adult cardiac surgical patients in the Society of Thoracic Surgeons database reporting on an association between the intraoperative use of cerebral oximetry monitoring and reduced major postoperative morbidity and mortality. Even though the causality was not established in this report, the authors called for a large prospective randomised controlled trial [21]. Clearly, it is not the monitor itself that can change the outcome but rather the therapeutic manoeuvres that are instituted based on the information provided by the cerebral regional oxygen saturation data. Management of restoration of rSO_2_ during cardiac surgery fundamentally differs from non-cardiac surgery, as the initial interventions in cardiac surgery are aimed towards reducing mechanical obstruction of venous return from the brain by direct surgical manipulation of the superior vena cava cannula and manipulation of the heart to optimize cerebral blood flow.

It is interesting to note that while pulse oximetry is considered to be a standard of care for patients undergoing any surgical procedure, cerebral oximetry seems to be held to a much higher standard, preventing its routine use in clinical practice, yet the success of either of these monitors has not been supported by scientific proof of an outcome benefit after either cardiac or non-cardiac surgery.

A small prospective study by Casati et al. found an association between decreased rSO_2_ during surgery and delay in recovery room discharge, increased risk of postoperative complications, and prolonged hospital length of stay in patients undergoing major abdominal surgery [8,16]. Furthermore, Morimoto et al. showed a relationship between preoperative regional cerebral oxygen saturation and the incidence of postoperative delirium in a small cohort of non-cardiac surgical patients [15].

Previously, we reported that low baseline of rSO_2_ (<50%) was associated with higher rates of postoperative delirium in a cardiac surgical population [19]. In addition Heringlake et al. showed that low rSO_2_ baseline levels were associated with increased major organ morbidity and mortality after cardiac surgery [13]. These reports were contrary to the findings of Baehner et al., who determined that preoperative rSO_2_ was not associated with postoperative complications in patients undergoing high-risk noncardiac surgery. The authors speculated that the discriminatory power of cerebral oximetry may have been insufficient due to individual variability of rSO_2_ values and confounding factors [22]. Unfortunately, we could not explore this relationship in the current study because only two patients displayed baseline rSO_2_ values below 50%.

An important future application of cerebral oximetry would be to personalize each individual patient’s blood pressure control based on their individual cerebral autoregulation curve. This would allow for a more personalised tailored approach to blood pressure management intraoperatively, thus representing the physiological needs of an individual patient as defined by their “true” lower and “true” upper limits of cerebral autoregulation. Intraoperative impairment of cerebrovascular autoregulation has been associated with increased postoperative morbidity and mortality after both cardiac and major non-cardiac surgery [23,24,25].

The main limitation of the current study is the small sample size. We did not reach the intended sample size of 394 patients, and this may have skewed our results towards non-significance. Currently, it remains unclear whether the restoration of rSO_2_ to baseline values results in improved perioperative morbidity and mortality after major non-cardiac surgery. Based on our results, we cannot recommend the use of cerebral oximetry as a standard of care for patients undergoing major non-cardiac surgery, nor can we determine which component of the algorithm is most influential. Future studies need to be adequately powered to determine the appropriate utility of intraoperative cerebral oximetry in patients undergoing major non-cardiac surgery.

## 5. Conclusions

In major non-cardiac surgery, cerebral desaturation is prevalent in over 85% of patients. Algorithm-guided therapy could restore rSO_2_, which may reduce postoperative morbidity. However, due to early determination of this study, future studies need to confirm the utility of cerebral oximetry.

## Figures and Tables

**Table 1:** Baseline demographic data and surgical characteristics.

Variable	Intervention Group	Control Group
	(*n* = 52)	(*n* = 49)
Age, mean ± SD, years	70 ± 5	69 ± 6
Male gender, *n* (%)	25 (48)	27 (55)
Height, cm	164 ± 17	167 ± 12
Weight, kg	73 ± 23	77 ± 19
Creatinine, mean ± SD, μM	82 ± 27	86 ± 37
Haemoglobin, mean ± SD, g/dL	134 ± 19	135 ± 17
**Type of surgery, *n* (%)**		
Gynaecology	10 (19)	8 (17)
Urology	10 (19)	12 (25)
General Surgery	7 (14)	5 (10)
Orthopaedics	6 (11.5)	6 (12)
Plastics	1 (2)	4 (8)
Vascular	10 (19)	7 (14)
Head and Neck	2 (4)	2 (4)
Liver	6 (11.5)	5 (10)
Duration of surgery, mean ± SD, min	362 ± 148	328 ± 117
Blood product transfusion, *n* (%)	5 (10)	4 (8)
Fluid balance, mean ± SD, ml	2229 ± 1352	1913 ± 895
Vasoactive drugs, *n* (%)	13 (25)	12 (24)
**Past Medical History, *n* (%)**		
Diabetes mellitus	12 (23)	10 (20)
Coronary artery disease	7 (14)	6 (12)
Hypertension	34 (65)	29 (59)
Cerebrovascular accident/transient		
Ischemic attack	3 (6)	4 (8)
Peripheral vascular disease	8 (15)	6 (12)
Chronic obstructive pulmonary disease	5 (10)	4 (8)
Chronic renal disease	4 (8)	3 (6)
Myocardial infarction	4 (8)	4 (8)
Thyroid disease	5 (10)	5 (10)
Atrial fibrillation	1 (2)	2 (4)
Alcohol > 20 units per week	5 (10)	5 (10)
Smoking history	18 (35)	15 (30)
**Medications, *n* (%)**		
Beta-blockers	10 (19)	9 (18)
Angiotensin-converting enzyme inhibitors	15 (29)	13 (26)
Calcium channel blockers	9 (17)	10 (20)
Aspirin	18 (35)	18 (37)
Statins	30 (58)	25 (51)
Antidepressants	2 (4)	3 (6)
**Functional scores, median [range]** Frailty scale	2.5 [1, 5]	3 [1, 7]
Duke Activity Status Index	24 [5, 51]	21 [7, 58]

**Table 2:** Incidence and duration of cerebral desaturation during the intraoperative period.

Interventional Group (*n* = 52)			Control Group (*n* = 49)	
	*Left Side*	*Right Side*	*Left Side*	*Right Side*
Baseline rSO_2_ (%)	62.5 [52, 84]	63 [56, 73]	64 [52, 82]	63 [43, 79]
Number of patients with rSO_2_ reduction ≥ 10% from baseline, *n* (%)	34 (65)	32 (62)	35 (71)	34 (69)
Bilateral	30 (58)		31 (63)	
Maximum reduction of rSO_2_ (%)	12 [0, 35]	12 [0, 61]	15 [0, 65]	12 [0, 85]
Number of episodes of rSO_2_ reduction ≥ 10% from baseline	1 [0, 7]	1 [0, 6]	2 [0, 8]	2 [0, 7]
Bilateral	2.5 [0, 10]		4 [0, 15]	
Duration of rSO_2_ reduction ≥ 10% from baseline, min	5.5 [0, 128]	4 [0, 30]	12 [0, 240]	7 [0, 364]
Bilateral	5 [0, 128] *		9 [0, 364]	

Data expressed as number (%) and median [range]. rSO_2_, regional cerebral oxygen saturation. * *p* = 0.01.

**Table 3:** Postoperative morbidity and mortality, functional outcomes, and hospital length of stay.

Variable, *n* %	Intervention Group (*n* = 52)	Control Group (*n* = 49)
Myocardial infarction	4 (8)	3 (6)
Atrial fibrillation	2 (4)	2 (4)
Delirium	1 (2)	2 (4)
Stroke/transient ischemic attack	1 (2)	0
Renal failure	2 (4)	2 (4)
Pulmonary embolism	1 (2)	0
Sepsis	1 (2)	1 (2)
Pneumonia	1 (2)	2 (4)
Wound infection	2 (4)	2 (4)
Reintubation	1 (2)	1 (2)
Return to operating room within 24 h	1 (2)	1 (2)
Length of hospital stay, days [range]	3 [1, 16]	3 [1, 14]
30-day mortality	0	0
Quality of Recovery-15, *n* [range]		
At baseline	127 [99, 150]	124 [48, 148]
At postoperative day 1	105 [54, 145] *	108 [64, 127] *
At hospital discharge	110 [44, 145] *	111 [64, 135] *
World Health Organization Disability Assessment Schedule, *n* [range]	4 [1, 23]	4 [1, 34]

* *p* < 0.03.

## Data Availability

Data are unavailable due to privacy or ethical restrictions.

## Abbreviations

The following abbreviations are used in this manuscript:

rSO_2_	Regional cerebral oxygen saturation
RCT	Randomised controlled trial
QoR15	Quality of Recovery-15
DFS	Disability Free Survival
WHODAS	World Health Organization Disability Assessment
